# The urban-rural disparities and factors associated with the utilization of public health services among diabetes patients in China

**DOI:** 10.1186/s12889-023-17198-y

**Published:** 2023-11-20

**Authors:** Xingli Ma, Wenyu Fan, Xindan Zhang, Shilong Zhang, Xia Feng, Suhang Song, Haipeng Wang

**Affiliations:** 1 Center for Health Management and Policy Research, School of Public Health, Cheeloo College of Medicine, Shandong University, Jinan, China; 2 NHC Key Laboratory of Health Economics and Policy Research(Shandong University), Shandong University, Jinan, China; 3 Department of Health Policy and Management, College of Public Health, University of Georgia, Athens, GA USA

**Keywords:** Public health service, Urban-rural disparities, Health education, Diabetes patients

## Abstract

**Background:**

Basic public health services for diabetes play an essential role in controlling glycemia in patients with diabetes. This study was conducted to understand the urban-rural disparities in the utilization of basic public health services for people with diabetes and the factors influencing them.

**Methods:**

The data were obtained from the 2018 China Health and Retirement Longitudinal Study (CHARLS) with 2976 diabetes patients. Chi-square tests were used to examine the disparities in the utilization of diabetes physical examination and health education between urban and rural areas. Logistic regression was performed to explore the factors associated with the utilization of diabetes public health services.

**Results:**

Among all participants, 8.4% used diabetes physical examination in the past year, and 28.4% used diabetes health education services. A significant association with age (OR = 0.64, 95% CI:0.49–0.85; P < 0.05) was found between patients’ use of health education services. Compared with diabetes patients living in an urban area, diabetes patients living in a rural area used less diabetes health education. (χ2= 92.39, P < 0.05). Patients’ self-reported health status (OR = 2.04, CI:1.24–3.35; P < 0.05) and the use of glucose control (OR = 9.33, CI:6.61–13.16; P < 0.05) were significantly positively associated with the utilization of diabetes physical examination. Patients with higher education levels were more likely to use various kinds of health education services than their peers with lower education levels (OR = 1.64, CI:1.21–2.22; P < 0.05).

**Conclusion:**

Overall, urban-rural disparities in the utilization of public health services existed. Vulnerable with diabetes, such as those in rural areas, are less available to use diabetes public health services. Providing convenient health service infrastructure facilitates the utilization of basic public health services for diabetes in older patients with diabetes, especially in rural areas.

## Introduction

The epidemic of diabetes mellitus and its complications has become one of the major health challenges around the globe [1]. According to the International Diabetes Federation (IDF), 537 million adults had diabetes in 2021, and this number is predicted to reach 634 million by 2030 and 783 million by 2045 [2]. China is one of the countries with the highest prevalence of diabetes in the world [3]; about 140 million people suffered from the disease in 2021. As a severe, complex and lifelong chronic disease, diabetes mellitus requires long-term care and treatment, and is one of the top leading causes of death and disability [4], resulting in enormous economic and social burdens on society and individuals [5, 6]. Diabetes has also become one of the major public health issues in China [7–9], hence, the management of diabetes intervention is of great importance [8].

The management of diabetes includes not only the guidance of medication usage but also a variety of comprehensive management strategies, such as lifestyle management [10]. The Chinese government launched the National Basic Public Health Services Program (EBPHS) in 2009 [11], including the management of patients with diabetes as an important component. Diabetes patients are managed by community healthcare providers and medical staff, and public health services are provided for free [12]. Basic public health services include physical examination, regular follow-up visits, and health education for patients with diabetes [12]. Diabetes health education services contain regular exercise, diet, weight control, smoking control, and alcohol restriction [13, 14].

Previous studies confirmed that diabetes management services and health education are the most cost-effective ways to control diabetes [15–17]. Individuals with diabetes self-management knowledge or higher health literacy are more likely to prevent diabetes better [18, 19]. Older patients with diabetes are more likely to have more health risk factors [13]. Numerous studies have assessed the effects of diabetes health education services on diabetes control and prevention [20–22]. Most studies on diabetes management focused on diabetes patients in specific regions, and most of the participants were managed by community health centers [23, 24].

A study has shown that disadvantaged people with diabetes in rural China underutilize health services [25]. Living in a rural area may experience a particular barrier to diabetes self-management, including costs, transport problems and limited health service access [26]. Compared to urban areas, rural patients in China have limited access to diabetes services and education resources [27]. In addition, patients living in rural areas have a lower level of awareness and ability to manage their diabetes [28], but few studies have investigated the factors associated with urban-rural disparities in the utilization of diabetes public health services. Identifying such factors may assist in improving diabetes public health services in rural areas.

Existing studies have highlighted that strengthening diabetes management and improving health education methods can increase patients’ awareness of self-management [19, 29] and improve their health status [30]. Thus, this study aimed to examine the utilization of diabetes public health services, and to explore the factors associated with such utilization in diabetes patients. Findings from this study may offer support in improving the delivery of diabetes public health services for people with diabetes, and better achieve primary and secondary prevention of diabetes to enhance glycemic control in people with diabetes.

## Methods

### Data source

Data for this study were obtained from the China Health and Retirement Longitudinal Study (CHARLS), 2018 wave. The CHARLS, conducted by Peking University, is a publicly available interdisciplinary survey (http://charls.pku.edu.cn/gy/gyxm.htm) with adults aged over 45 years old in China [31]. The 2018 CHARLS data has a high response rate of 85%, and the data are nationally representative of high-quality [32].

CHARLS used a multi-stage stratified probability-proportional-to-size sampling method and face-to-face interviews with respondents [32]. The interview covered several aspects, such as basic information, health status, financial status, medical expenses and insurance [32]. By the time the national follow-up was completed in 2018, its sample had covered 19,000 respondents in 12,400 households [31]. The investigation was conducted in accordance with the guidelines of the Declaration of Helsinki and was approved by the Peking University Ethics Review Board, and all participants signed a written informed consent form [31].

### Participants and definition

The diabetes patients were defined as (1) self-reported being diagnosed with diabetes or elevated blood glucose (including abnormal glucose tolerance and elevated fasting blood glucose, or (2) having a history of diabetes or elevated blood glucose during the last visit.

We retain patients who answered “yes” to the question “Was diagnosed with diabetes or hyperglycemia by a doctor?“ Patients who responded “yes” and those selected in the past as having the disease of diabetes, i.e., excluding non-diabetic patients. To understand the current status of diabetes public health services in China in recent years, urban-rural disparities, and their risk factors, this study restricted the sample to patients with diabetes in the latest CHARLS data. Finally, a total of 2976 diabetic patients were included in the analyses.

### Measurements

In this study, diabetes public health services were defined as diabetes physical examination and health education. In CHARLS, participants were asked if they have used diabetes physical examination or health education in the past year. Among them, the diabetes physical examination is “Have you had diabetes physical examination by doctors during the last year?”, the answer is “yes” or “no”. Health education is “Have your care providers ever given you health education/advice on the following?”, and the options include weight control, diet, exercise, and smoking control.

Covariates include individual demographic (gender, age, marital status), socioeconomic characteristics (education level, residence, medical insurance, social activity), and health status (self-reported health, blood glucose control), Among them, Age was divided into 45–59 years old group, 60–75 years old group and 75 years old and above group. Education level was divided into four groups-illiterate/semiliterate, primary school, junior high school, and senior high school or above. Marital status was classified into three groups-married and living with a spouse, married but not living with a spouse, and single. Glucose control is a measure of how seriously patients take glucose monitoring, in terms of whether they are clear about their blood glucose status. Patient glucose control was measured by three options: blood glucose is under control, blood glucose is not under control, and not sure. Self-reported health was divided into three groups-good, bad or fair. The types of medical insurance are divided into urban and rural resident medical insurance, urban employee medical insurance and other types.

### Statistical analyses

Descriptive statistics on public health services utilization with diabetes were expressed in frequency and percentage. The chi-square test was applied to estimate urban-rural differences in the use of public health services for people with diabetes and its differences between different populations. Logistic regression models were performed to analyze the factors of diabetes public health services utilization. Statistically significance was considered as P ≤ 0.05.

## Results

### Basic characteristics of the participants and urban-rural disparities

The characteristics of the participants are shown in Table 1. Among the 2976 participants included in the analysis, 79.2% lived in rural areas. About half of the patients (51.5%) were between the ages of 60 and 75. With respect to education level, 56.9% were illiterate/semiliterate,19.9% had primary school education, 15.0% had secondary school education, and 8.2% had senior high school and higher education. The majority (72.9%) of the participants were married and living with a spouse. Only 14.2% of patients reported living in good health status. 82.7% of people are using urban and rural resident medical insurance. In addition, 64.7% of the participants considered their glycemic control to be bad. Participants from urban had better health status and higher blood glucose control levels than those from rural areas.

**Table 1:** Basic characteristics of the participants and urban-rural disparities

Characteristics	Total(N = 2976) [n (%)]	Urban(N = 588) [n (%)]	Rural(N = 2360) [n (%)]	χ2	P
**Gender**					
Male	1302(43.7)	274(46.6)	1010(42.8)	2.74	0.098
Female	1674(56.3)	314(53.4)	1349(57.2)		
**Age**					
45–59	822(27.6)	187(31.9)	615(26.1)	9.46	0.009
60–75	1534(51.5)	295(50.3)	1231(52.2)		
75-	619(20.8)	105(17.9)	513(21.7)		
**Self-reported health status**					
Good	383(14.2)	92(17.3)	285(13.3)	20.21	< 0.001
Fair	1181(43.8)	260(49.0)	913(42.5)		
Bad	1131(42.0)	179(33.7)	950(44.2)		
**Blood glucose control**					
No	1926(64.7)	288(49.0)	1632(69.2)	90.55	< 0.001
Yes	883(29.7)	263(44.7)	598(25.3)		
Don’t know	167(5.6)	37(6.3)	129(5.5)		
**Education level**					
Illiterate/semiliterate	1694(56.9)	187(31.8)	1504(63.8)	342.31	< 0.001
Primary school	591(19.9)	122(20.7)	462(19.6)		
Junior high school	448(15.1)	146(24.8)	299(12.7)		
Senior high school and higher	243(8.2)	133(22.6)	94(4.0)		
**Marital status**					
Married and living with a spouse	2170(72.9)	445(75.7)	1699(72.0)	5.54	0.063
Married but not living with a spouse	171(5.7)	37(6.3)	131(5.6)		
Single	635(21.3)	106(18.0)	529(22.4)		
**Medical insurance**					
Urban employee medical insurance	370(12.4)	244(41.5)	109(4.6)	607.57	< 0.001
Urban and rural resident medical insurance	2460(82.7)	328(55.8)	2120(89.9)		
Other medical insurance	146(4.9)	16(2.7)	130(5.5)		
**Social activity**					
Yes	1425(47.9)	341(58.2)	1067(45.3)	31.50	< 0.001
No	1547(52.1)	245(41.8)	1291(54.7)		

### Diabetes public health services utilization and urban-rural disparities

Table 2 shows the utilization of public health services and urban-rural disparities among diabetes patients in China. 91.6% had not been examined for diabetes in the past year, indicating a low utilization of public health services by people with diabetes. 71.6% of the patients had not used any diabetes health education. Urban-rural disparities in diabetes health education existed as well. Compared with urban diabetic patients, rural diabetic patients have poorer access to diabetes health education programs (χ2= 92.39, P < 0.05).

**Table 2:** Public health services utilization and urban-rural disparities among diabetes patients

	Total [n (%)]	Urban [n (%)]	Rural [n (%)]	χ2	P
**Physical examination**					
Yes	249(8.4)	50(8.5)	195(8.3)	0.04	0.852
No	2727(91.6)	538(91.5)	2164(91.7)		
**A type of health education**					
Yes	846(28.4)	258(43.9)	566(24.0)	92.39	< 0.001
No	2130(71.6)	330(56.1)	1793(76.0)		
**Four types of health education**					
Yes	228(7.7)	77(13.1)	144(6.1)	33.16	< 0.001
No	2748(92.3)	511(86.9)	2215(93.9)		
**Health education on weight**					
Yes	458(15.4)	159(27.0)	285(12.1)	82.31	< 0.001
No	2518(84.6)	429(73.0)	2074(87.9)		
**Health education on exercise**					
Yes	606(20.4)	218(37.1)	369(15.6)	135.55	< 0.001
No	2370(79.6)	370(62.9)	1990(84.4)		
**Health education on diet**					
Yes	777(26.1)	235(40.0)	522(22.1)	78.46	< 0.001
No	2199(73.9)	353(60.0)	1837(77.9)		
**Health education on smoking**					
Yes	338(11.4)	102(17.3)	225(9.5)	29.10	< 0.001
No	2638(88.6)	486(82.7)	2134(90.5)		

Notes: A type of health education refers to the patient has received at least one of the four types of health education (weight, exercise, diet, smoking). Four types of health education refer to patients having received all of the four types of health education (weight, exercise, diet, and smoking). Health education on weight: whether the patient had received health education on weight control; health education on exercise: whether the patient had received health education on exercise; health education on diet: whether the patient has received health education on diet; health education on smoking: whether the patient has received health education on smoking cessation

### Diabetes public health services utilization by population groups

Table 3 shows the comparison of diabetes public health service utilization by different groups of diabetes patients. Patient age was associated with the use of basic public health services (P < 0.05). Compared with patients living in better health status, patients living in worse health status have poorer access to diabetes health education programs (P < 0.05). Among patients who had been examined for diabetes in the past year, 71.1% had good glucose control (P < 0.05) and as many as 81.9% got married and were living with their spouse (P < 0.05). Patients’ glucose control, education level, marital status, health insurance, place of residence, and social activity are associated with patient access to relevant health education. For example, Patients with better glucose control were more likely to go on to use health education on weight control than those who were unsure whether their blood glucose was under control (p < 0.05).

**Table 3:** Diabetes public health services utilization by the characteristics of participants

Characteristics	Physical examination [n (%)]	Health education on weight[n (%)]	Health education on exercise[n (%)]	Health education on diet[n (%)]	Health education on smoking[n (%)]
**Gender**					
Male	100(7.7)	215(16.5)	270(20.7)	320(24.6)	232(17.8) **
Female	149(8.9)	243(14.5)	336(20.1)	457(27.3)	106(6.3)
**Age**					
45–59	70(28.1) **	187(40.8) **	232(38.3) **	287(36.9) **	150(44.4)**
60–75	148(59.4)	220(48.0)	306(50.5)	396(51.0)	148(43.8)
75-	31(12.4)	51(11.1)	68(11.2)	94(12.1)	40(11.8)
**Self-reported health status**					
Good	23(6.0) **	53(12.7)	64(11.5)	84(11.7)	46(14.8)
Fair	89(7.5)	189(45.4)	254(45.5)	327(45.7)	149(47.9)
Bad	120(10.6)	174(41.8)	240(43.0)	304(42.5)	116(37.3)
**Blood glucose control**					
No	54(21.7) **	76(16.6) **	107(17.7) **	133(17.1) **	52(15.4) **
Yes	177(71.1)	348(76.0)	447(73.8)	567(73.0)	261(77.2)
Don’t know	18(7.2)	34(7.4)	52(8.6)	77(9.9)	25(7.4)
**Education level**					
Illiterate/semiliterate	128(51.4) *	164(35.8) **	224(37.0) **	331(42.6) **	117(34.6) **
Primary school	47(18.9)	99(21.6)	143(23.6)	177(22.8)	73(21.6)
Junior high school	53(21.3)	112(24.5)	137(22.6)	159(20.5)	80(23.7)
Senior high school and higher	21(8.4)	83(18.1)	102(16.8)	110(14.2)	68(20.1)
**Marital status**					
Married and living with a spouse	204(81.9) **	365(79.7) **	492(81.2) **	621(79.9) **	283(83.7) **
Married but not living with a spouse	10(4.0)	26(5.7)	31(5.1)	43(5.5)	21(6.2)
Single	35(14.1)	67(14.6)	83(13.7)	113(14.5)	34(10.1)
**Medical insurance**					
Urban employee medical insurance	32(12.9)	112(24.5) **	154(25.4) **	170(21.9) **	89(26.3) **
Urban and rural resident medical insurance	210(84.3)	341(74.5)	437(72.1)	589(75.8)	243(71.9)
Other medical insurance	7(2.8)	5(1.1)	15(2.5)	18(2.3)	6(1.8)
**Residence**					
Urban	50(20.4)	159(35.8) **	218(37.1) **	235(31.0) **	102(31.2) **
Rural	195(79.6)	285(64.2)	369(62.9)	522(69.0)	225(68.8)
**Social activity**					
Yes	130(52.2)	273(59.6) **	358(59.2) **	442(57.0) **	201(59.5) **
No	119(47.8)	185(40.4)	247(40.8)	334(43.0)	137(40.5)

Notes: Health education on weight: whether the patient had received health education on weight control; health education on exercise: whether the patient had received health education on exercise; health education on diet: whether the patient has received health education on diet; health education on smoking: whether the patient has received health education on smoking cessation. Blood glucose control: patients’ knowledge of their own glucose control; *P ≤ 0.05; **P ≤ 0.01

### The factors associated with the utilization of Diabetes public health services

The factors associated with public health service utilization are shown in Table 4. Diabetes patients reporting poor health status were more favorably examined for diabetes than those reporting good health status (OR = 2.04, 95%CI:1.24–3.35; P < 0.05). Compared with participants with uncontrolled blood glucose, those with controlled blood glucose used more diabetes physical examination (OR = 9.33,95%CI:6.61–13.16; P < 0.05).

**Table 4:** The factors associated with the utilization of diabetes public health services by logistic regression models

	Diabetes examination	Weight control health education	Exercise health education	Diet health education	Smoking control health education
	OR (95%CI)	OR (95%CI)	OR (95%CI)	OR (95%CI)	OR (95%CI)
**Gender (ref.=male)**					
Female	1.14(0.83–1.56)	1.06(0.81–1.38)	1.31*(1.02–1.68)	1.62**(1.27–2.07)	0.28**(0.21–0.38)
**Age(ref.=45–59)**					
60–75	1.30(0.92–1.84)	0.64**(0.49–0.85)	0.75*(0.57–0.97)	0.74*(0.57–0.96)	0.52**(0.38–0.71)
75-	0.94(0.55–1.59)	0.43**(0.28–0.68)	0.49**(0.32–0.74)	0.51**(0.34–0.75)	0.51**(0.32–0.83)
**Self-reported health status(ref.=good)**					
Fair	1.19(0.72–1.97)	1.27(0.86–1.88)	1.54*(1.06–2.23)	1.51* (1.06–2.15)	1.04(0.69–1.58)
Bad	2.04**(1.24–3.35)	1.83**(1.22–2.75)	2.39**(1.62–3.51)	2.14**(1.48–3.09)	1.31(0.85–2.02)
**Education level (ref.= Illiterate)**					
Primary school	0.89(0.60–1.33)	1.39(0.99–1.94)	1.64**(1.21–2.22)	1.49**(1.10–2.01)	1.03(0.71–1.51)
Junior high school	1.48(0.98–2.24)	2.10**(1.48–2.97)	1.83**(1.31–2.56)	1.57**(1.12–2.18)	1.30(0.89–1.92)
Senior high school and higher	1.02(0.56–1.88)	2.50**(1.60–3.92)	2.16**(1.40–3.34)	1.68*(1.08–2.60)	1.44(0.89–2.33)
**Marital status (ref.= Married and living with a spouse)**					
Married but not living with a spouse	0.58(0.28–1.19)	0.68(0.40–1.15)	0.58*(0.35–0.96)	0.65(0.40–1.04)	0.64(0.36–1.15)
Single	0.79(0.52–1.21)	1.01(0.69–1.45)	0.77(0.54–1.08)	0.75(0.54–1.04)	0.64(0.40–1.03)
**Residence(ref.=urban)**					
Rural	1.39(0.93–2.08)	0.69*(0.51–0.93)	0.55**(0.41–0.74)	0.77(0.57–1.04)	1.06(0.74–1.51)
**Medical insurance (ref.=Urban employee medical insurance)**					
Urban and rural resident	1.13(0.69–1.85)	0.89(0.61–1.29)	0.71(0.50–1.02)	0.73(0.51–1.05)	0.67*(0.45–0.99)
Other medical insurance	1.01(0.40–2.55)	0.24*(0.08–0.71)	0.63(0.30–1.30)	0.45*(0.22–0.92)	0.32*(0.11–0.89)
**Blood glucose control(ref.=no)**					
Yes	9.33**(6.61–13.16)	14.35**(10.66–19.32)	16.13**(12.42–20.95)	23.80**(18.57–30.50)	14.93**(10.53–21.15)
Don’t know	4.10**(2.28–7.39)	5.67**(3.54–9.07)	6.63**(4.38–10.04)	11.09**(7.60–16.20)	6.56**(3.80-11.31)
**Social activity(ref.=no)**					
Yes	1.06(0.79–1.43)	1.14(0.88–1.47)	1.20(0.95–1.52)	1.17(0.93–1.47)	1.08(0.81–1.45)

Notes: OR, Odds ratio; 95%CI, 95% confidence intervals. Weight control: weight control health education; exercise: exercise health education; diet: diet health education; smoking control: smoking control health education. Ref.: reference category. Blood glucose control: patients’ knowledge of their own glucose control; *P ≤ 0.05; **P ≤ 0.01

Patients aged 60 years and above (OR = 0.64, 95%CI:0.49–0.85; P < 0.05) made less use of health education services compared to those aged 45–59 years. Patients living in bad health status used more health education on weight control (OR = 1.83,95%CI:1.22–2.75; P < 0.05), exercise (OR = 2.39,95%CI:1.62–3.51; P < 0.05) and diet (OR = 2.14,95%CI:1.48–3.09; P < 0.05) than those living in good health. Compared to patients who were married and living with their spouse, patients who were married but not living with their spouse used even less health education on exercise (OR = 0.58,95%CI:0.35–0.96; P < 0.05). Compared to those with low levels of education, highly educated patients use more weight health education (OR = 2.10, 95%CI:1.48–2.97; P < 0.05), exercise health education (OR = 1.64, 95%CI:1.21–2.22; P < 0.05), and diet education (OR = 1.49, 95%CI:1.10–2.01; P < 0.05) services. In addition, blood glucose control was positively associated with receiving basic public health services (P < 0.05).

## Discussion

This study demonstrates the utilization of public health services for people with diabetes in China. We found that only a few patients used diabetes physical examinations and health education in the past year. Urban-rural disparities in the use of diabetes health education existed, with a high rate of use of diabetes health education in urban areas. The study examined the association between patients’ utilization of public health services and participants’ characteristics. Rural and lower socioeconomic status diabetes patients are at a disadvantage in terms of utilization of basic public health services. Across all age groups, patients between the ages of 60 and 75 years participated more in basic public health services. Patients’ utilization of public health services for diabetes was related to their self-reported health, patient attention to blood glucose monitoring, education level, marital status, and social activities.

Our study found that rural areas had more patients with poor health status and worse access to diabetes health education, which is consistent with previous studies [33]. This demonstrates the urgent need for improved diabetes health education in rural China by increasing access to diabetes education programs for those with poor health status. This study also found that rural patients were less likely to use health education on all types of diabetes than their urban counterparts. The reasons for the urban-rural disparities remain unclear. One of the potential reasons lies in the inequitable allocation of basic community public health services facilities between rural and urban areas leading to the lack of easy access to diabetes education programs in rural areas [34]. Another reason may also be due to the fact that urban patients have more awareness of health education programs [17], compared to rural patients [35], since rural diabetic patients have poor health literacy [36]. Technology and mHealth can be used in the future to effectively reduce barriers to rural diabetes self-management and improve health outcomes [37, 38]. The study found that the proportion of the population using education programs on tobacco control was lower in rural areas than in urban areas, which a previous study demonstrated already [39]. The phenomenon may be explained by the fact that people of poor socioeconomic status are at greater risk of developing tobacco dependence [40]. In addition, rural patients may have a lower awareness of the health risks associated with smoking [41]and therefore place less emphasis on tobacco control education. Provide specific training for diabetes educators and medical advisors to prioritize tobacco use issues [42].

Self-reported health status has usually been considered as a valid and reliable measure [43]. In our analysis, health status (self-reported health, glycemic control) is associated with the utilization of diabetic public health services. This suggests that good self-health can produce motivating behaviors to improve diabetes, which is consistent with previous studies. [44]. This study found that compared with patients with good self-perceived health status, patients with poor self-perceived health status are more willing to use diabetes health examination and education. This may be because, according to the Health Belief Model [45], poor health beliefs promote behavioral changes in patients, so that they may actively seek public health services for diabetes [46]. This study also found that, compared with patients with uncontrolled blood sugar, patients with better blood sugar control use more health education, this may be because participating in health education allows patients to better control their blood glucose [19]. At the same time, poor glycemic control or relapse will make patients unwilling to continue to use health education [47]. Therefore, diabetes health educators can provide support to patients in the form of personalized counseling and telehealth sessions to help them build confidence to cope with the disease and overcome their fears [48].

Our study found a low use rate of diabetes physical examination and health education among poorly educated patients, compared to those who were more educated. This result aligns with other studies [49]. This may result from the fact that a higher education levels can improve diabetes awareness [50], and patients with higher education levels are more willing to accept diabetes education services. Meanwhile, from the aspect of health education, women with diabetes used more exercise and diet education, compared to men with diabetes. Existing evidence shows that women are known to have more awareness of diabetes than men [51] and that women are more willing to control their blood glucose levels [50]. Older patients over the age of 75 participated the least in basic public health services, probably because the older patients were in poor health and had difficulty using public health services [52]. With frail older patients already, long distances and transportation are barriers to older adults using public health services [53]. This guides the home health care efforts we should promote for people with physically inconvenient diabetes [35]. Older patients have limited knowledge and can easily overlook health issues [53] or fail to understand the professional messages of health service providers [54]. This requires healthcare providers to provide easy-to-understand forms of service delivery, such as one-on-one counseling, so that older patients can understand and implement these services well [55].

Globally, it is well known that public health service utilization of diabetes is below expectation [56, 57], this is consistent with our study. Especially in some low- and middle-income countries (LMICs) with poor health care systems [58], the burden of diabetes is not affordable for diabetes patients and their families. The lower utilization of basic public health services makes patients’ self-management literacy and ability poor, thus failing to meet the requirements of blood glucose control and endangering their health. China has the most significant number of people with diabetes in the world, yet in rural China, the quality of basic public health services for diabetes is poor [59]. Thus, evidence from China on the utilization of basic public health services for diabetes may be generalized to LMICs among others, and further contribute to improving global control of diabetes. Our study results may shed light on practical insights for increasing the use of diabetes physical examination and education in other developing countries.

This study is subject to several limitations. First, as a cross-sectional study, causal relationships are not able to be identified. Second, although the selected data were well controlled, there may be recall bias may exist because many of the questions were self-reported by patients. Third, CHARLS obtains data from middle-aged and older adults in China and the results are more difficult to extrapolate to young people. Last, the factors associated with the use of public health services were limited to the demand-side perspective and did not include those associated with the supply side.

## Conclusion

Diabetes patients with low socio-economic status, such as those in rural areas or those with low education levels used less diabetes public health services. Urban-rural disparities in the utilization of diabetes health education and the utilization were affected by education level and health status. Providing convenient health service infrastructure facilitates the utilization of basic public health services for diabetes in older patients with diabetes, especially in rural areas. Also, Health education services in the form of personalized counseling are more likely to attract vulnerable populations with diabetes to utilize health education programs.

## Data Availability

The datasets used in this study are available in the China Health and Retirement Longitudinal Study repository, [http://charls.pku.edu.cn/zh-CN].

## Abbreviations

CHARLS: China Health and Retirement Longitudinal Study
IDF: International Diabetes Federation
EBPHS: National Basic Public Health Services Program
LMICs: Low- and middle-income countries
